# A qualitative study of how psychiatrists change their practice and approach to work effectively in Indigenous communities

**DOI:** 10.1192/j.eurpsy.2026.11451

**Published:** 2026-07-31

**Authors:** L. Mehl-Madrona, B. J. Mainguy

**Affiliations:** 1 Family Medicine, University of New England, Portland; 2 Research, Coyote Institute, Orono; 3 Psychiatry, Northern Light Acadia Psychiatry Residency, Bangor; 4 Philosophy, University of Maine; 5 Psychotherapy, Maine Street Medicine Works, LLC; 6 Education, Coyote Institute, Orono, United States

## Abstract

**Introduction:**

We had heard from psychiatrists that acceptance by Indigenous community members requires susbstantial alterations from how they were taught to practice in residency. Acceptance increases efficacy. We wanted to learn in a more formal sense what those changes were.

**Objectives:**

We aimed to understand how psychiatrists change their approach to be accepted and to be effective when working within Indignenous communities.

**Methods:**

Twenty-nine participants were found through convenience sampling at psychiatric meetings from their presentations, from interest groups, and from word or mouth. A focused, narrative-style interview was conducted with questions designed to elicit how the psychiatrists had changed their approach and practice to be accepted by Indigenous community members. Constructivist grounded theory methods were used to analyze the interviews for recurrent themes. No personal data was collected or stored.

**Results:**

The following themes were common:

1. Reduction of professional distancing
2. Comfort with dual relationships
3. Accepting and offering gifts
4. Willingness to meet people in non-office settings (homes, stores, tribal offices, street corners, boats, cars, other vehicles)
5. Acceptance of others’ world views and experiences as valid without argument or dismissal.
6. Respectful curiosity about the Others’ culture.
7. Willingness to be present and helpful at community events and to help (setting tables, washing dishes, making coffee)
8. Willingness to attend ceremonies and other events when invited.
9. Learning a different rhythm, speed, and style of communication
10. Psychiatrists who refuse to make changes in their style of practice tend to be avoided by community members and tend not to enjoy their visits to the communities, and tended to stop visiting these communities.

**Conclusions:**

Conventional psychiatric training is grounded in Eurocentric, mainstream cultural values and styles of interacting which are not necessarily shared by Indigenous communities. Successful engagement requires the development of cultural literacy sufficient to avoid standing out as too different or unusual. Doctors may find themselves working with clinic staff as clients or their driver to and from the airport or tribal leaders who pay their salary. More disclosure of personal stories facilitates engagement and requires careful filtering of stories that do not contain present personal issues and still are funny or engaging. Therapeutic encounters are not limited to office settings. Being willing to be helpful in non-medical ways goes far in building trust and engagement. A client’s saying that he can’t take medication because caribou meat makes his blood different is accepted and not met with argument and leads into discussion about what can people like him do. The ultimate acceptance is to be invited to attend ceremonies and community events. Refusing to attend can destroy hard won trust.

**Disclosure of Interest:**

None Declared

